# Up close and real: living and learning in a remote community builds students’ cultural capabilities and understanding of health disparities

**DOI:** 10.1186/s12939-017-0615-x

**Published:** 2017-07-06

**Authors:** Rosalie D Thackrah, Maeva Hall, Kathryn Fitzgerald, Sandra C Thompson

**Affiliations:** Western Australian Centre for Rural Health, University of Western Australia, Perth, Western Australia Australia

**Keywords:** Aboriginal health, Allied health professionals, Interprofessional education, Remote health care delivery, Health disparities, Service learning

## Abstract

**Background:**

Rural and remote communities in Australia fare worse than their urban counterparts across major health indicators, with geographic isolation, restricted accessibility to health services, socioeconomic disadvantage, lifestyle and behavioural factors all implicated in poorer health outcomes. Health disparities, which are especially stark in Australian Aboriginal and Torres Strait Islander populations, underscore the urgent need to build a culturally responsive and respectful rural health workforce.

Allied health student placements in settings with high Aboriginal populations provide opportunities for the development of cultural capabilities and observation of the causes and impact of health disparities. A service learning pedagogy underpinned by strong campus-community partnerships can contribute to effective situated learning. Positive placement experiences can also encourage future rural practice alleviating workforce shortages. This article reports on the first stage of a proposed longitudinal investigation into the impact of remote placements on clinical practice and employment choices.

**Methods:**

In-depth interviews were undertaken with health science students and recent graduates from Australian universities who spent up to 4 weeks at the remote community of Mt. Magnet (Badimaya country) in Western Australia. Interviews, which occurred between two and 12 months following the placement were recorded, transcribed and thematically analysed for patterns of meaning.

**Results:**

Factors which contributed to positive professional, personal and socially responsive learning experiences were identified. These included pre-placement cultural training to build understanding of the local Aboriginal community, peer support, community engagement, cultural exchanges and interprofessional collaboration. Highlights were associated with relationship-building in the community and opportunities to apply insights into Aboriginal cultural ways to clinical and community practice. The role of the Aboriginal mentor was integral to students’ understanding of the social and cultural dynamics in the practice setting. Challenges related to the logistics of supervision in remote locations and workloads.

**Conclusions:**

The interprofessional placement offered students a unique opportunity to experience how isolation, socioeconomic disadvantage and cultural factors conspire to produce health inequities in remote Australian settings and to observe how communities respond to their circumstances. Despite difficulties encountered, learnings derived from the application of clinical, social and interprofessional skills, and rural employment opportunities that arose following graduation, were all highly valued.

## Background

### Rural and remote communities: contributing factors to health disparities

Well documented workforce shortages and health disparities in Australian rural and remote communities, especially in Aboriginal^1^ populations, led to the establishment of University Departments of Rural Health and Rural Clinical Schools, and the rise of rural health professional organisations [1–3]. Rural or “outer regional” communities are defined as settlements beyond metropolitan centres, whereas “remote” communities are characterised by “small and highly dispersed populations, higher proportions of Indigenous people, and less access to all services” [4]. Recent rural classifications differentiate “outer regional” areas using population size and distance from nearest major towns as a measure of “rurality” or “remoteness”. This is important as classification of rurality is tied to workforce incentives, which have not always reflected the challenges associated with attracting health professionals to small rural towns [5].

Australian rural and remote communities fare worse than their urban counterparts across all major health indicators. Poorer health outcomes are associated with geographic isolation, restricted accessibility to health services, socioeconomic disadvantage, and lifestyle and behavioural factors [2, 4, 6]. Maldistribution of health services is a contributing factor to health disparities, with recruitment and retention of the rural health workforce a recognised barrier to care [2, 6]. As noted by Greenhill and colleagues, with reference to medical practitioners and specialists, “the more remote the community, the more likely it is to be underserved” [3].

Restricted access to services associated with geographic isolation is further exacerbated by socioeconomic disadvantage experienced by many remote community members. While less than 5% of Australia’s population live in remote settings, for historical and cultural reasons, Aboriginal Australians represent a greater proportion of the population with increasing remoteness [4]. Living conditions and social circumstances for these populations are more likely to be sub-standard; overcrowded housing, high rates of unemployment, low levels of educational attainment and poor health outcomes have been extensively documented [4, 7, 8]. Investment in the early years of life has been identified as a key starting point to reduce health inequities that arise from socioeconomic disadvantage [9]. Allied health professionals can play a vital role in this regard, however, access (excluding Aboriginal Health Workers) is limited in remote communities; these professionals comprise the lowest proportion of the rural health workforce per 100,000 population yet remote communities usually have greater needs [2].

### Building the rural health care workforce

“Allied health professional” is a term that covers multiple disciplines of health workers, excluding medical, nursing and dental professionals. The definition of an allied health professional on the Allied Health Professionals Australia website includes a practitioner who has: completed a university level program with appropriate accreditation; a direct patient care role; articulated competency standards; representation by a national professional organisation; a defined scope of practice, and robust and enforceable regulatory mechanisms [10]. Social workers, physiotherapists, occupational therapists, speech pathologists, psychologists and pharmacists are included as allied health professionals, although this list is not exhaustive [2].

Challenges associated with attracting and retaining health professionals in rural and remote areas have been widely documented, including initiatives to bolster the rural health workforce [2, 3, 6, 11]. Some strategies such as the pipeline approach, aim to foster interest in the health professions among rural secondary students in the expectation that they will practise rurally [2]. Medical schools have expanded rural clinical training over the last decade and more than a third of students now undertake at least 1 year of clinical practice in rural settings [3]. While this has increased the number of medical graduates interested in rural practice [3], rural career uptake by allied health professionals is similarly needed to address workforce shortages and community needs. Governments have also funded rural allied health training but are seeking evidence that demonstrates the benefit of their investment as to date there is a dearth of research which links initiatives with anticipated outcomes [12]. This study, which focuses on student experiences and learnings, highlights the importance of longitudinal studies into remote clinical placements and their impact on future employment choices.

### Developing cultural capabilities and interprofessional collaboration

Closely aligned with building the rural health workforce is the preparation of health professionals to deliver culturally respectful care to Aboriginal communities. Considerable progress has been made since the 2004 Committee of Deans of Australasian Medical Schools (CDAMS) report, which championed Aboriginal content and Aboriginal involvement in curriculum design and delivery [13]. The concept of cultural capabilities, now widely used in curriculum frameworks, refers to “skills, knowledge and behaviours that are required to plan, support, improve and deliver services in a culturally respectful and appropriate manner” [14]. Most health professional programs now incorporate mandatory Aboriginal content and many offer clinical placements to facilitate the development and application of cultural capabilities through interactions with Aboriginal community members [15–17].

Cultural immersion experiences in remote Aboriginal communities can have a profound effect on student learning. Numerous studies have highlighted the power of the “lived experience”: the humility and respect derived from sustained interaction with Aboriginal people; the insights gained into Aboriginal belief systems and cultural protocols; and deeper awareness of the impact of social and economic disadvantage on health outcomes [18–20]. Most reported studies, however, are discipline specific. Few have investigated whether similar benefits accrue to students who undertake placements interprofessionally and if collaboration adds value to the learning experience acquired.

Collaborative learning and team-based work practices underpin interprofessional education (IPE), which is now widely promoted in university training programs in recognition of complex care needs and the importance of integrated approaches [21, 22]. Interprofessional learning has been defined as “the acquisition of interprofessional knowledge, skills and attitudes which students would not usually acquire effectively through other processes” [21]. Typically, two or more health professionals learn together in classes or teams and come to understand the roles of other team members. While enhanced professionalism, teamwork and collegiality are desirable outcomes of interprofessional education, the ultimate goal is improved health service delivery resulting in better health outcomes for individuals and communities [21–23].

### Models of learning in situ

Pedagogical frameworks associated with “experiential” and “situated learning” are relevant to clinical placements designed to provide cultural immersion and/or team-based practice experiences. Experiential learning derives from individuals’ emotional responses to experiences and meaning attached to those experiences in a setting [24], whereas a “situated learning” framework emphasises acquisition of knowledge in a learning context or a “community of practice” [25]. Both approaches associate effective learning with students’ ability to reflect on their responses to a setting and extract personal and professional meaning from their experiences [26, 27].

While the focus of these approaches is upon the nature of learning, they are compatible with and frequently incorporated into a service learning model, which engages students in activities that benefit community stakeholders and have direct academic learning outcomes [28, 29]. Service learning is effective in the development of socially responsive knowledge where the goal is to expose students to social issues and give them the experience and skills to respond to those issues [28]. Civic engagement, when underpinned by successful campus-community partnerships, can heighten students’ awareness of community strengthens and capacities and encourage socially responsive behaviours through collaboration [28, 30]. Throughout the learning process, however, opportunities to reflect on experiences are paramount. Despite placements occurring with community consent and collaboration, risks remain that unintended harm may occur; reflective sessions and/or interactive journal writing may help limit these risks [18, 31].

### The Mt. Magnet placement

The Mt. Magnet placement commenced at the Western Australian Centre for Rural Health (WACRH), University of Western Australia in 2013. WACRH has its main office located in Geraldton, a large regional centre in the Midwest of Western Australia. While the value of cultural immersion experiences and interprofessional learning underpinned the aims of the placement, of equal importance was the prospect that students may consider rural or remote practice as a consequence of their experience. An appreciation of the strengths and challenges in rural and remote communities, especially those with substantial Aboriginal populations, is fundamental to the preparation of socially responsive and culturally respectful health professionals and to encouraging future rural practice [19, 20]. Attributes that can be acquired in rural and remote placements include problem solving, independence, resilience, cultural respect, and a heightened awareness of the link between socioeconomic circumstances and health outcomes [20]. There is also potential for on-going connections with communities through future employment.

Mt. Magnet is located 342 km east of Geraldton and 560 km north-east of Perth, and provides services to pastoral and mining industries in the region [32]. In the 2011 census, Mt. Magnet had a population of 532 people, of whom 43.5% (230) identified as Aboriginal Australians [33]. Local Aboriginal people identify as Badimaya and are located within Yamatji country, which encompasses the Murchison and Gascoyne regions of Western Australia. The traditional language, also known as Badimaya, is endangered, although revitalisation efforts are on-going [34].

Mt. Magnet was chosen as the preferred location for the program as WACRH had a longstanding relationship with an Aboriginal Elder in the community. The township had been identified as a high needs area where student programs could contribute to improved health in the community; the Elder encouraged this involvement. A house had been purchased in the town and refurbished as accommodation for students and staff. Long-term relationships with other community members also existed including with the cultural mentor, who was employed by WACRH.

Clinical practice and learning was primarily focussed on children at the primary school, however students were also expected to connect with community organisations and contribute to local events. Students were asked to keep a reflective journal, which was utilised weekly by their supervisor to help ascertain their adjustment, learning and any challenges they experienced while on the placement.

## Methods

The study utilised in-depth interviews, a technique which is appropriate when the population is small and rich, experiential and reflective data is required [35]. The questions, which were developed by the lead researcher in consultation with academic staff involved in the placement, were designed with two groups of participants in mind: students completing their program and those who had recently commenced employment. In addition to gathering data on the placement experience, information on factors influencing employment choices was sought from recent graduates.

Standard interview schedules were developed by the research team. Interviews took place in 2014 and 2015, between two and 12 months following the placement. The lead researcher (RDT), who was not involved in the arrangement of placements, teaching or assessment of students, conducted all of the interviews. Most were between 45 and 90 min in length and the majority were conducted by telephone due to participants being in widely dispersed geographic locations. Interviews conducted face-to-face were held at the University of Western Australia or at the interviewer’s home nearby. All interviewees agreed to be contacted again for future follow-up as part of the longitudinal study.

Interviews were recorded and transcribed. Transcriptions were completed by a professional transcription service and recordings erased eight days after completion. Allocation of codes ensured confidentially and all transcripts were stored in a locked filing cabinet. Original files were stored on a password-protected computer. Transcripts were thematically analysed by the lead researcher in consultation with the research team using standard techniques: immersion in data through multiple readings; annotation, initial and axial coding; identification and categorisation of emerging themes; bracketing of significant quotations; and refinement of themes as patterns of meaning and repetition of those patterns were discerned [35]. The generation of a thematic map, selection of quotations that captured the diversity of responses, interpretation based on a pedagogical framework associated with situated learning theory [25, 27] and discussion within the research team, all contributed to the analysis of the findings. Ethics approval for the study was granted by the Human Research Ethics Office of the University of Western Australia. Interviewees completed and retained copies of the Participant Consent and Participant Information Forms. Questions about the study were addressed prior to the commencement of interviews.

## Results

The potential subjects for this research comprised 24 students who completed the placement in 2013–14. All were invited to participate in the study, however, eight did not respond to e-mail requests or phone messages, and four declined due to work or family constraints. Of the 12 interviewees, ten completed a full semester placement, which included time spent in Geraldton and Mt. Magnet, while two, who were part of the initial pilot program, completed the Mt. Magnet placement only.

### Participant characteristics

The age range of participants was 22–30 years, with two-thirds (8) under the age of 25 years; 11 of the 12 participants were single, and only two were male. All were Australian-born, with the majority from Western Australia (9); two were born in New South Wales and one in Queensland; 50% (6) were raised in rural areas. Over half (7) identified by heritage as Australian (Australian-born parents), while five had one Australian-born parent with the other born in one of the following countries: New Zealand, South Africa, England, Ireland and Austria. Participants came from five health science programs including speech pathology (4); occupational therapy (4); social work (2); exercise physiology (1); and a generalist health science degree (1). Ten participants completed a semester placement based in Geraldton, which included between two to 4 weeks at Mt. Magnet, while two completed a 5 week placement based in Mt. Magnet only. At the time of interview, eight were employed with six of these based in a range of regional and rural areas including Kalgoorlie, Alice Springs, Geraldton and Lismore. Four participants were in the final or penultimate year of their program at the time of interview.

### Preparation and motivation for undertaking placement

Despite the fact that participants came from a number of universities and a range of programs, all had received some instruction in Aboriginal health and cultures, although the nature of the content and its timing in programs varied. All participants considered the knowledge acquired to be valuable, but emphasised the importance of additional content provided by WACRH, especially the “Understanding Yamatji” workshop delivered by a local Aboriginal staff member. The workshop was singled out by all participants as vital to their preparation for working in the region. *“The cultural training we did with xxx, our cultural mentor*. *.*. *was the best that I could ever receive. He went into communication differences, body language and silences, all important stuff”.* Most participants felt they were adequately prepared for the placement from a cultural perspective, but as one noted, *“I only realised when I was there that I was pretty ill-prepared from a practice point of view”.* A distinction was often made between cultural and clinical preparation as participants had little experience in the application of cultural knowledge in clinical settings.

Motivations for undertaking the placement varied. One participant highlighted the importance of a 6 month volunteering opportunity in South Africa undertaken in a gap year; how it shaped her thinking and influenced her decision to apply for the placement.

It opened my eyes up to a whole new world . . . like the concept of race and health and wealth . . . and the inequalities due to race, and when I came back to Australia I could see that in my own backyard. . . so that was a big push for why I wanted to go rural, and especially out to Mt. Magnet.

Some offered pragmatic reasons for considering the placement: accommodation costs were covered; a concentrated placement reduced clinical demands later in the year; and *“it will look good on my CV”.* Most, however, identified the attraction of a rural lifestyle and working with Aboriginal children at Mt. Magnet as big drawcards.

### Reflections on the Mt. Magnet placement

#### Logistics

Students were provided with accommodation at Geraldton and Mt. Magnet at no charge, and access to a vehicle for the three and a half-hour journey between the settings. A shared house (students and staff) was provided at Mt. Magnet and all those interviewed were happy with the arrangement. Occasionally, if it was a full house, study space was tight, but in general, participants identified the benefits of communal living and also appreciated having the cultural mentor living on site. Children frequently gathered around the house (a source of delight) but if they over-stayed their welcome, the cultural mentor intervened and his authority was respected. Heidi the goat and Lady Cackles the chicken lived in the backyard. *“I think it made it a bit more homely*. *.*. *and because we told the kids about them, every day they would be like, ‘how’s the goat, can we come and visit’ and it really helped us connect with them”.* No participants expressed concern about their safety, with most making reference to the sense of security provided by the cultural mentor. Occasionally internet connections failed, which was a source of anxiety for those who were homesick or required to submit assessments.

#### Clinical and peer support

Clinical supervisors were readily available to students by telephone and e-mail as they were not residents of Mt. Magnet. The supervisors scheduled visits to meet the needs of the students, with visits throughout and greater local supervisory oversight at the beginning of the placement; their stays were generally of one or two nights. Some participants considered this inadequate while others suggested that the independence derived helped build their confidence.

I know a couple of people . . . would have liked more OT (occupational therapy) support out there. I quite enjoyed the independence and just having a go myself. I think I built on my skills a lot more . . . if there was someone there I would have just asked them what to do. I definitely got better as an OT because of that experience.

A number of participants expressed a fear of failing the placement:

Basically I was scared of failing. We’d heard a lot of things that we shouldn’t be doing while in the community and it left me with a feeling of doing something wrong and upsetting people. Simple things which might be offensive in their culture . . . it’s the fear of the unknown as well, just not knowing what you are in for.

Difficulties encountered were often neutralised by peer support. Those who completed the placement with friends considered it a big advantage. *“Going with a friend was huge. Without my friend there, I don’t think we would have lasted, to be honest. You needed that support, definitely. So without her it would have been very different and a lot harder”.* Peer support was valuable in clinical practice and as an emotional buffer. Some participants were very confronted by the living conditions and social problems in the community and others lacked confidence in clinical settings. *“When we were struggling we would always try to get together and brainstorm and, you know, we kind of built good connections with each other then, clinically and personally, and that really helped”.*

#### Cultural exchanges and socially responsive learnings

Numerous opportunities to build relationships with local Aboriginal people and enhance understanding of social and cultural issues in the community were identified. Most students spent time at Bidi Bidi, a local service set up to support Aboriginal mothers and grandmothers, and learned about community dynamics, including the impact of social disadvantage and restricted health service delivery in remote settings. The cultural mentor also facilitated opportunities at weekends for students to explore the surrounding country, learn about cultural protocols and sites of significance, eat traditional foods and meet local community members. These opportunities were embraced, however, participants were aware of being guests “on country” and acknowledged that time was needed to establish trust and build relationships.

Involvement in the morning bus run and school breakfast club also provided insights into social and living conditions, which were impediments to regular school attendance. One participant observed that these activities *“showed how it takes a whole community to help get that child to school*. *.*. *that was enlightening”.* The bus driver, who was known to families, encouraged the children to get ready and delivered them to the breakfast club, where Aboriginal and Islander Education Officers (AIEO) supported by some Aboriginal parents provided nourishing food to start the day. This local initiative was a response to complex family dynamics arising from social disadvantage, and aimed to support parents and encourage school attendance. It was understood that children missed school for many reasons apart from illness. Involvement in the program enabled students to establish rapport with children, contextualise their circumstances and develop a heightened awareness of the impact of social disadvantage on community members. These insights were valuable in social and clinical interactions with children.

#### Interprofessional education (IPE)

Participants were positive about the benefits that accrued from interprofessional collaboration and identified IPE as a unique feature of the placement. *“I think it was extremely valuable and a good approach to assessment and treatment. It was really good to maximise sessions and attain joint goals, and it just made therapy very enriched”.* Value was attached to understanding different professional roles and perspectives, clinical collaboration in assessments and interdisciplinary team work, although the latter was viewed by some as underdeveloped. One participant drew attention to tensions that can arise when roles are not clearly defined.

I definitely agree that IPE has a place in learning environments because it prepares you for real clinical situations where you might be working within a team for a client at a hospital or out-patients service . . . but it only works well when you are very clear on your roles and what you are supposed to be doing together. Issues around role clarity made it (IPE) a bit more challenging in a remote environment.

The majority of students had experienced IPE in Geraldton where their supervisors were readily available. Although not all placements at Mt. Magnet had an IPE component (several placements attracted students from only one health discipline), IPE clearly presented challenges for some students. Others revelled in the experience:

As an ex phys (exercise physiology student) I was with a speech pathologist and a health promotion girl. And it was a fantastic bonding experience . . . it helped me learn how to work interprofessionally . . . there was quite a lot we could do with health promotion to help them out, but there was a lot we could do with speech pathology as well. It is important to know how you fit into that interdisciplinary team.

Another participant noted that despite initial anxiety about working interprofessionally on the placement, *“now that I’m working I can see how much it actually helped me. I’m in a multidisciplinary team and have a much deeper understanding of the skills of others in my team”.* Some participants also noted that IPE experience gained on the placement was cited in job applications, as interdisciplinary team work was considered to be highly valued in the workplace.

#### Highlights, challenges and key learnings

Highlights of the clinical placement were largely associated with community engagement, cultural learnings and the application of these to clinical practice. The cultural mentor and the local children were central to positive accounts of the placement and closely connected to important learning outcomes. For example, one participant noted with reference to the cultural mentor that:

He was not only a friend, but like a grandfather to us, such a valuable resource talking to us about language and different values, and introducing us to his lifestyle and friends. We would sometimes just sing songs and watch the fire crackle of an evening and learn about cultural stuff . . . the connection with nature, and with no one talking constantly, and no need to fill the gaps in conversation . . . just being bathed in that simplicity was amazing.

Another participant noted that that highlights were *“definitely around building relationships”* and for another it was about *“being part of the community”.* Working and interacting with the local children after school hours was identified as a rewarding experience. This was also related to the freedoms attached to a rural lifestyle. “*I just loved that lifestyle. I know there is some bad stuff that goes on in such a small town, but it’s so nice to see the kids playing in the streets and just going off to the creek to catch frogs. I absolutely loved that”.*

The children also presented challenges, which sometimes became learning opportunities. A speech pathology student reflected on how she was forced to re-think clinical strategies.

From a clinical aspect I had to re-think a lot of ways that I would do things. Having kids that were not happy to sit down and do a task meant I needed to incorporate whole body movements, such as running round the classroom picking up items, rather than just getting them to shuffle cards. It really did challenge the way I would do practice, but it was such an enriching experience.

Challenges cited by most participants related to supervision and workloads. Those who had little paediatric experience in clinical placements often lacked confidence in their ability to work independently with children, and requested more direct supervision, while others felt they benefited from the independent decision-making required. All participants considered the workload to be heavy and for some this was a source of considerable stress. *“We were up at 6.30am to go to the breakfast club and we wouldn’t go to be bed until 11pm at night after doing paperwork and prep. It was very intense*. *.*. *but at the same time it was a really good experience, one of the best experiences I’ve had”.*

Key learnings centred on the provision of services in remote Aboriginal communities. Most participants noted that their thinking about health care delivery changed as a result of the placement. They witnessed inequities in resource allocation that characterise isolated communities and learned how isolation, socioeconomic disadvantage and cultural factors conspire to produce poorer health outcomes. They learned the importance of building strong community connections and developing cultural insights that enhance communication in clinical practice. *“You really need to have a holistic outlook with your client . . . to learn about their beliefs and values before you try and do health care. I should have known this before but I didn’t”.* Another commented that *“it allowed me to learn more about different ways of practising, like narrative therapy. So I’ve become more interested in these techniques because Aboriginal people say ‘it works well with us’”.* Key learnings also derived from opportunities to talk with Aboriginal people who were directly affected by the “Stolen Generation”^2^ and through observing cultural protocols associated with death and dying.

#### Impact on future practice settings and program feedback

Although most participants noted that they were drawn to a rural lifestyle and many were raised in rural settings, the Mt. Magnet placement reaffirmed the benefits of working rurally. All students drew attention to tight labour market conditions, which meant few employment opportunities in metropolitan settings, but despite this most desired rural practice. The experience *“sort of confirmed that ‘yeah, I can do it*. *.*. *and what’s more, I like it’”.* Another commented that *“I already wanted to work rurally, so that influenced my decision to do the prac, which probably influenced my decision to then work rurally”.* It was suggested that the placement provided sound preparation for rural practice, removed uncertainties about what to expect, and enhanced confidence about being able to cope. Rural employment was also associated with more generalised caseloads, considered beneficial to new graduates. As noted earlier, six of the eight employed participants were working in regional and rural areas and most identified the placement experience as valuable in securing a position soon after graduation. Only one participant expressed an interest in working long-term in remote settings; most acknowledged they would have difficulty with the isolation. To some extent, this reflects the age of the participants, but also highlights the on-going difficulties associated with retention of health professionals in remote locations.

All those involved in these initial placements were invited to provide feedback to WACRH and suggest areas for improvement. Positive feedback in this study was common; all participants recommended the placement with some suggesting it be made compulsory. Others noted, however, that it was only in retrospect that they realised its enormous value as a learning experience, especially when they commenced employment. The workload was considered to be too heavy by all participants; adjustments were made as a result of this feedback. Others recommended that the placement be undertaken with a friend as support offered by friends was invaluable during times of stress, uncertainty or ill-health.

## Discussion

Three themes emerged from the findings: the socio-cultural and geographic authenticity associated with the experience; the importance of community connections established; and the application of learnings to clinical practice. These themes draw together the development of students’ cultural capabilities with their understanding of health disparities in remote, disadvantaged communities and vindicate investment in remote clinical training programs that aim to build the rural health care workforce.

### Authenticity

Immersion experiences are associated with health professional students gaining insights into the lived experience of community members and recognising their agency in health care delivery. In a study that assessed the impact of a rural community immersion experience on students’ understanding of primary health care principles, Playford and Lines [36] found that students’

definition of health broadened; they grasped the practical meaning of ‘access’, recognising that not all people are equally served in a community; and their views on ‘community’ as a target of programs shifted so they understood the necessary role of the community in effecting its own sustainable health care programs.

It has also been observed that rich learning experiences can arise from immersion in settings where different cultural beliefs and practices are encountered, provided that students are well prepared in advance and given adequate support throughout the placement [18–20]. The Mt. Magnet placement on Badimaya country introduced students to the realities of living in a geographically isolated community with a substantial Aboriginal population. They interacted with disadvantaged families and observed the impact of health disparities on local communities, especially the children.

For students, remoteness meant vast distances, restricted direct availability of supervisors, intermittent internet connections and an inability to return home at weekends. In these circumstances, students supported one another, drew upon their preparation and training to cope with difficulties that arose, and developed independence and interprofessional capabilities most thought beyond them at that time. This occurred in a cross-cultural context that demanded sensitive deciphering of cultural messages and the establishment of trust and rapport with community members. Learning that derives from actual encounters with Aboriginal people is more meaningful to students than content delivered in classrooms [16, 27]. The authenticity of the experience was emphasised by participants in this study and contributed to the uniqueness of the learning process, and its application to clinical practice.

### Community connections: social and cultural insights

Implicit in health promotion strategies and primary health care provision is the notion that health is optimised when individuals and communities have increased control over their circumstances and decision-making [37]. Engagement with communities and recognition of their inherent strengths are fundamental to successful health care delivery and improved health outcomes. Where cultural differences exist, students and new practitioners must explore ways to meaningfully engage with community members, but remain cognisant of their novice status [20]. A delicate balance exists between the dual aims of service learning: the achievement of academic learning outcomes and the provision of beneficial services to the community [28]. It has been suggested that as campus-community partnerships increase, questions related to the impact of student placements on communities, especially in disadvantaged and/or culturally diverse settings, will require more attention [38].

In this study, making community connections was pivotal to the success of the placement; the cultural mentor played an important role in this respect. At that time the mentor shared accommodation with students and this facilitated interactions with community members. While some participants expressed concern about causing offence, their heightened sensitivity reflects growing self-awareness. Most identified opportunities to interact with and learn from Aboriginal people as the highlights of the experience. Invitations to contribute to organisations and local events were embraced by students and welcomed by community members. These opportunities provided insights into community dynamics and cultural protocols, the extent of socioeconomic disadvantage and its impact on health and education, and provided a platform for relationship-building, which underpins successful clinical practice.

### Application to practice

Students modified practice strategies and reconsidered more limited concepts of health and well-being in response to cultural insights acquired during the placement. Opportunities to contextualise children’s circumstances through participation in the morning school bus run and breakfast club, the establishment of relationships with children outside the school environment, and deeper insights into cultural mores, including communication styles, resulted in more creative and productive interactions in clinical settings. Interprofessional learning opportunities provided students with novel ways to combine their skills and deepen their understanding of various professional roles. Although role clarity was identified as essential for successful collaboration, participants in this study, like others on interprofessional rural placements [22, 23], valued learning from one another. While some experienced discomfort when confronted with cultural differences and communication styles, these feelings mirror those expressed by Aboriginal people in unfamiliar settings, such as city hospitals, and have implications for future clinical practice [20, 39].

Experiential and situated learning theories, which focus on the learner in context and learning as a process derived from interactions with others, provide a useful framework to interpret the findings of this study. The authenticity of the immersion experience, the connections made with community members and interactions with staff and peers, all contributed to effective learning that was simultaneously applied in clinical settings. This occurred within the context of a service learning model, which encourages civic engagement and the development of socially responsive knowledge and behaviours [28].

### Limitations of the study

Of the 24 students involved in the placement, only 12 were interviewed. While experiences of all students were not captured, participants represented a cross-section of allied health professions. The study was also limited in scope given its focus upon the impact of the experience on student learning. The impact of the placement on community members, however, has not been explored in this study and so was observed only through students’ eyes.

## Conclusions

The Mt. Magnet placement offered students a unique opportunity to experience how isolation, socioeconomic disadvantage and cultural factors conspire to produce health inequities in remote Australian settings, and to observe how local communities respond to their circumstances to effect change. The placement allowed students to participate in these responses, deepening their understanding of the social and cultural determinants of Aboriginal health.

The placement and associated lifestyle were highly valued by those who participated in this study and reinforced a desire to work rurally. The authenticity attached to learning in-situ, and insights gained from community connections and interprofessional collaboration, resulted in more meaningful and creative practice, adapted to children’s circumstances and cultural background. Participants reported that this understanding had changed their thinking about practice and would influence how they approached consultations more broadly in the future. While difficulties were encountered, all participants recalled their experiences on Badimaya country as valuable, both personally and professionally, and encouraged others to “go rural”.

While this study offers a tentative link between positive rural placement experiences and future rural employment, longitudinal studies are required to build an evidence base in this area. Further research into the impact of service learning on remote communities is also required to ensure that mutual benefits are derived from placements and community needs prioritised.

## Footnotes

1 The Indigenous peoples of Australia include Aboriginal people and Torres Strait Islanders. In Western Australia, Torres Strait Islanders represent a very small proportion of Indigenous Australians and in this setting there is a preference for the use of the term Aboriginal over terms such as Indigenous or abbreviations such as ATSI. For the purpose of this paper in remote Western Australia, we respectfully use the term Aboriginal and for simplicity use this term to refer to the Aboriginal and Torres Strait Islander peoples of Australia. The term Indigenous appears in this paper when reference is made to literature using the term or when it is used in quotations.

2 The term “Stolen Generation” refers to Aboriginal and Torres Strait Islander children who were removed from their families during the early 1900s through to the late 1960s as a result of Australian Government policies of the day.
